# Post-Shingrix Vaccination Guillain-Barré Syndrome Presentation in the Emergency Department: A Case Report and Literature Review

**DOI:** 10.7759/cureus.77777

**Published:** 2025-01-21

**Authors:** Jan Drmota, Khandker S Quader, Salman Naeem

**Affiliations:** 1 Emergency Medicine, William Harvey Hospital, Ashford, GBR

**Keywords:** autoimmune, case report, guillain-barre syndrome, shingrix, vaccine, varicella-zoster

## Abstract

Guillain-Barré syndrome (GBS) is a rare neurological disorder of the peripheral nervous system. We present a case of 65-year-old female patient who developed GBS post-Shingrix inoculation. The patient, with no recognised GBS risk factors, presented with acute bilateral ascending lower limb peripheral nerve pathology eight days post-Shingrix vaccination. Other than abnormal lower limb neurological findings, her examination and biochemical results were normal. Imaging indicated possible inflammation in bilateral lumbosacral plexus. Treatment with IVIG achieved some symptom improvement, but she did not return to her baseline. Functionally, she was able to stand and mobilise 50 metres with a four-wheeled walker. Our case report highlights the importance of considering this syndrome as a differential in patients with no common GBS risk factors post-Shingrix vaccination, despite the large gap in the literature. Further studies into vaccine components as autoimmune triggers are necessary to minimise risk of GBS in the post-vaccination period.

## Introduction

Guillain-Barré syndrome (GBS) is a rare neurological disorder regarded as an acute inflammatory demyelinating polyradiculoneuropathy with varied presenting symptoms involving both sensory and motor peripheral nervous systems. Typically, it is characterised as a symmetrical ascending paralysis with reduced reflexes. The neurological deficit may extend and affect the cranial nerves, respiratory function and autonomic nervous system [1,2].

The aetiology of the disease is broadly understood to be subsequent to respiratory or gastrointestinal infections, particularly by Campylobacter jejuni infections, but vaccinations such as the influenza vaccine are also known factors. Global incidence of GBS is rising with current estimates being around 1.9 per 100,000 population. GBS is associated with increasing age and a 1.5:1 male-to-female incidence ratio [2,3].

While evidence-based protective benefits of vaccination programmes are established, long-term temporal analyses on larger populations expose unaccounted side-effects. For example, GBS has been identified as a potential side-effect risk associated with vaccinations such as influenza, polio, Ebola, Zika and COVID-19 vaccines [4,5]. The estimated risk of GBS post vaccination is minimal. For influenza vaccination, it is estimated to increase the incidence of GBS by one to three per million vaccine doses administered [6].

The hypothesised mechanism of GBS is based on molecular mimicry where the inflammatory response to a foreign antigen incorrectly triggers a response to structurally similar auto-antigens. Campylobacter jejuni expresses ganglioside-like structures (e.g. lipo-oligosaccharides). Vaccine components may similarly contain antigens structurally similar to neuronal gangliosides causing cross-reactivity. The influenza hemagglutinin is thought to induce an anti-GM1 auto-antibody response damaging axonal components in the peripheral nervous system and blocking nerve conduction [7].

The lack of robust diagnostic tests for GBS makes causative analysis difficult. Temporal associations with the Shingrix varicella zoster vaccine have been reported in four case reports [8-11]. The literature gap indicates further research into the potential cross-reactive risk of the common Shingrix vaccine components is necessary to reduce the disease burden. This case report describes a 65-year-old female patient who developed GBS post-Shingrix vaccination.

## Case presentation

A 65-year-old female patient presented to the emergency department with a one-day history of sudden onset ascending bilateral lower limb weakness and numbness in June 2024. She denied any recent illness, change in bowel motions, speech difficulty, or musculoskeletal injuries. She noted that on the morning prior to the onset of symptoms she was at her baseline of fully independent. Of note in her history was administration of the varicella zoster and pneumococcal vaccine eight days prior to presentation but with no immediate side-effects.

Her past medical history included hypothyroidism supplemented with levothyroxine. She had no known drug allergies. Her surgical history included being a kidney donor and a Hartmann’s procedure with end colostomy for perforated diverticulitis.

Her previous vaccination history is summarised in Table 1. She denied any previous vaccination-related complications.

**Table 1:** Summary of vaccination history.

Date	Vaccination name
March 2021	COVID Chadox1 AstraZenecca
May 2021	COVID Chadox1 AstraZenecca
September 2021	Influvac sub-unit Tetra vaccine Viatris
November 2021	Comiraty COVID-19 mRNA Pfizer
October 2022	Comiraty COVID-19 mRNA Pfizer
September 2023	Adjuvant quadrivalent influenza vaccine (surface antigen, inactivated) Seqirus
September 2023	Comiraty COVID-19 mRNA Pfizer

On examination, she was alert and oriented with no signs of respiratory distress. Her observations were stable with a respiratory rate of 18 breaths a minute, 99% saturations on room air, blood pressure of 173/93 mmHg, heart rate of 58 beats per minute and a temperature of 36.8 degrees Celsius.

Her respiratory, cardiovascular and abdominal examinations were normal.

Neurological examination of her lower limbs is summarised in Table 2.

**Table 2:** Summary of neurological examination of lower limbs. * Medical Research Council (MRC) Scale for Muscle Strength

	Left	Right
Examination	High-arched foot	High-arched foot
Gait	Unable to stand up independently.	
Tone	Normal	Normal
Reflexes		
Patellar	Diminished with re-enforcement	Diminished with re-enforcement
Ankle	Diminished with re-enforcement	Diminished with re-enforcement
Plantar	Equivocal	Flexor
Ankle clonus	Nil	Nil
Sensation		
Light touch	Reduced	Reduced
Pinprick sensation	Reduced	Reduced
Vibration sensation	Reduced	Reduced
Proprioception	Intact	Intact
Power*		
Hip flexion	3	2
Hip extension	3	3
Hip abduction	3	3
Hip adduction	3	3
Knee flexion	4	4
Knee extension	4	4
Ankle dorsiflexion	4	4
Ankle plantarflexion	4	4

Cranial nerves were intact and tone, sensation, power and reflexes were intact in her upper limbs. The Hoffmann test negative. Cerebellar signs were negative.

Blood investigations

Blood results identified no abnormalities. Her biochemical results are summarised in Table 3.

**Table 3:** Summary of patient’s biochemical results.

Blood test	Patient’s values	Reference range
Serum creatine	67 µmol/L	52.2 – 91.9 µmol/L
Estimated glomerular filtration rate	83 mL/min/1.73m^2^	> 90 mL/min/1.73m^2^
Sodium	138 mEq/L	135 – 145 mEq/L
Potassium	4.3 mEq/L	3.5 – 5.2 mEq/L
Chloride	104 mEq/L	96 – 106 mEq/L
Albumin-adjusted calcium	2.5 mmol/L	2.19 – 2.56 mmol/L
Magnesium	0.84 mmol/L	0.65 – 1.05 mmol/L
Creatine kinase	122 U/L	22 – 198 U/L
C-reactive protein	4 mg/L	< 5mg/L
Haemoglobin	144 g/L	120 – 150 g/L
White cell count	7.5 × 10^9^/L	2.6 – 11.0 × 10^9^/L
Lactate	1.1 mmol/L	0.5 – 2.2 mmol/L
Glucose	5.2 mmol/L	4 – 7 mmol/L
Serum vitamin B12	446 pg/mL	180 – 1000 pg/L
Folate	10 μg/L	4 – 10 μg/L
Thyroid-stimulating hormone	0.5 mU/L	0.5 – 5.0 mU/L
Free T4	14 μg/dL	4.8 – 12.7 μg/dL
Urea	4.4 mmol/L	2.5 – 7.8 mmol/L
CASPR2 antibody	Negative	Negative
LGI1 antibody	Negative	Negative
Gamma GT	21 U/L	5 – 40 U/L
Serum ACE	59 U/L	16 – 85 U/L
Rheumatoid factor	< 20 U/mL	< 20 U/mL
C ANCA	Positive with no antibodies to proteinase-3 or myeloperoxidase detected	Negative
C3	1.10 g/L	0.88 – 2.01 g/L
C4	0.28 g/L	0.16 – 0.48 g/L
Serum total protein	73 g/L	60 – 83 g/L
Globulin	30 g/L	20 – 35 g/L
IgG	11.0 g/L	6 – 16 g/L
IgA	1.4 g/L	0.8 – 3.0 g/L
IgM	0.93 g/L	0.4 – 2.5 g/L
Electrophoresis	No paraprotein detected	No paraprotein detected
Neuronal antibody screen (including Hu, Yo Ri, CV2/CRMPS, Sox-1, Zic4, Tr and Amphiphysin)	Negative	Negative
HbA1c	39 mmol/mol	< 42 mmol/mol
Cerebrospinal fluid		
CSF protein	0.26 g/L	0.18 – 0.58 g/L
CSF glucose	4.1 mmol/L	2.77 – 4.44 mmol/L
CSF white blood cell count	< 1 mm^3^	< 1 mm^3^

Imaging

**Figure 1:**
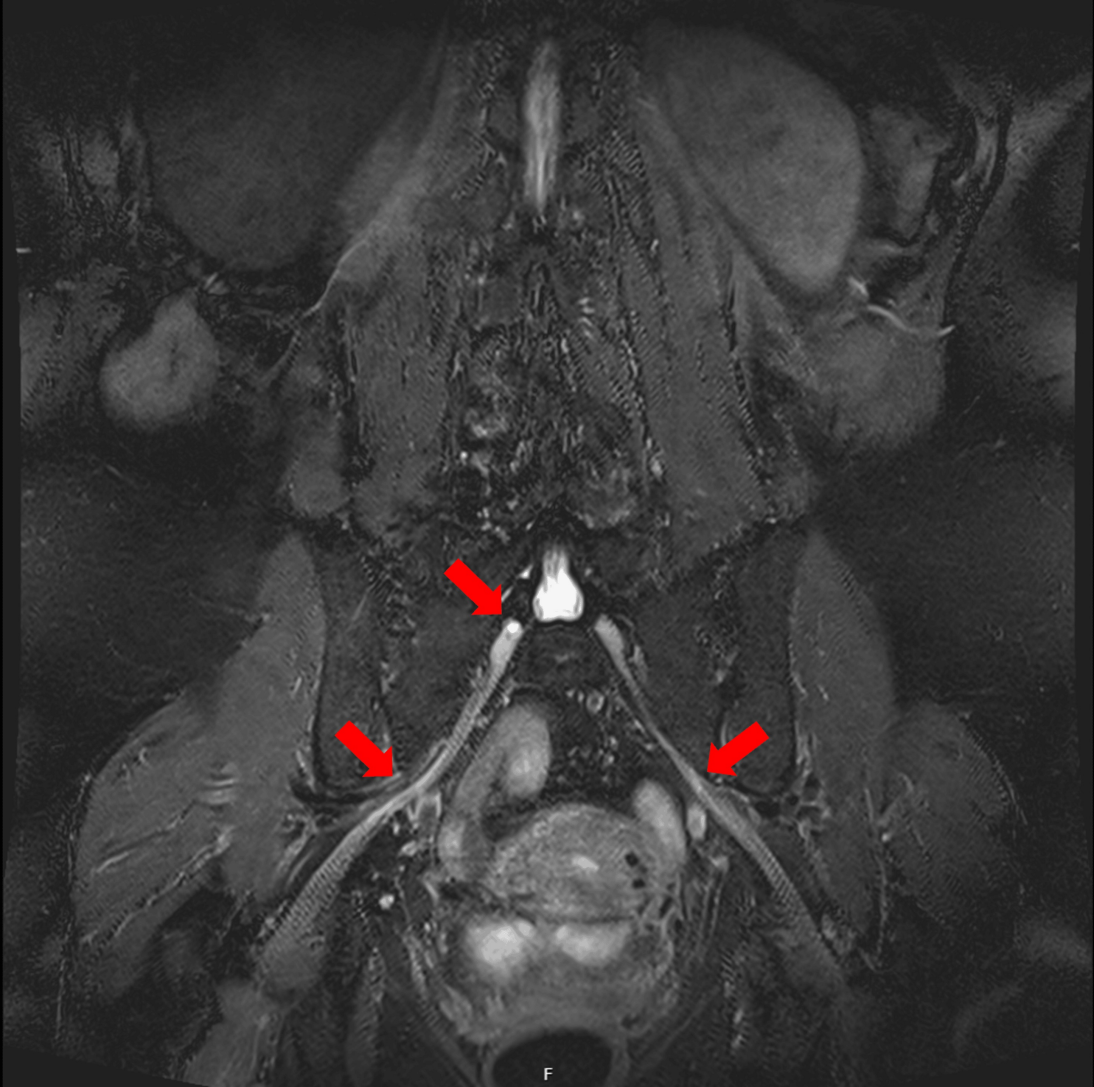
T2-weighted short tau inversion recovery (STIR) hyperintense signal highlighted with red arrows in bilateral lumbosacral plexus on coronal magnetic resonance imaging.

**Figure 2:**
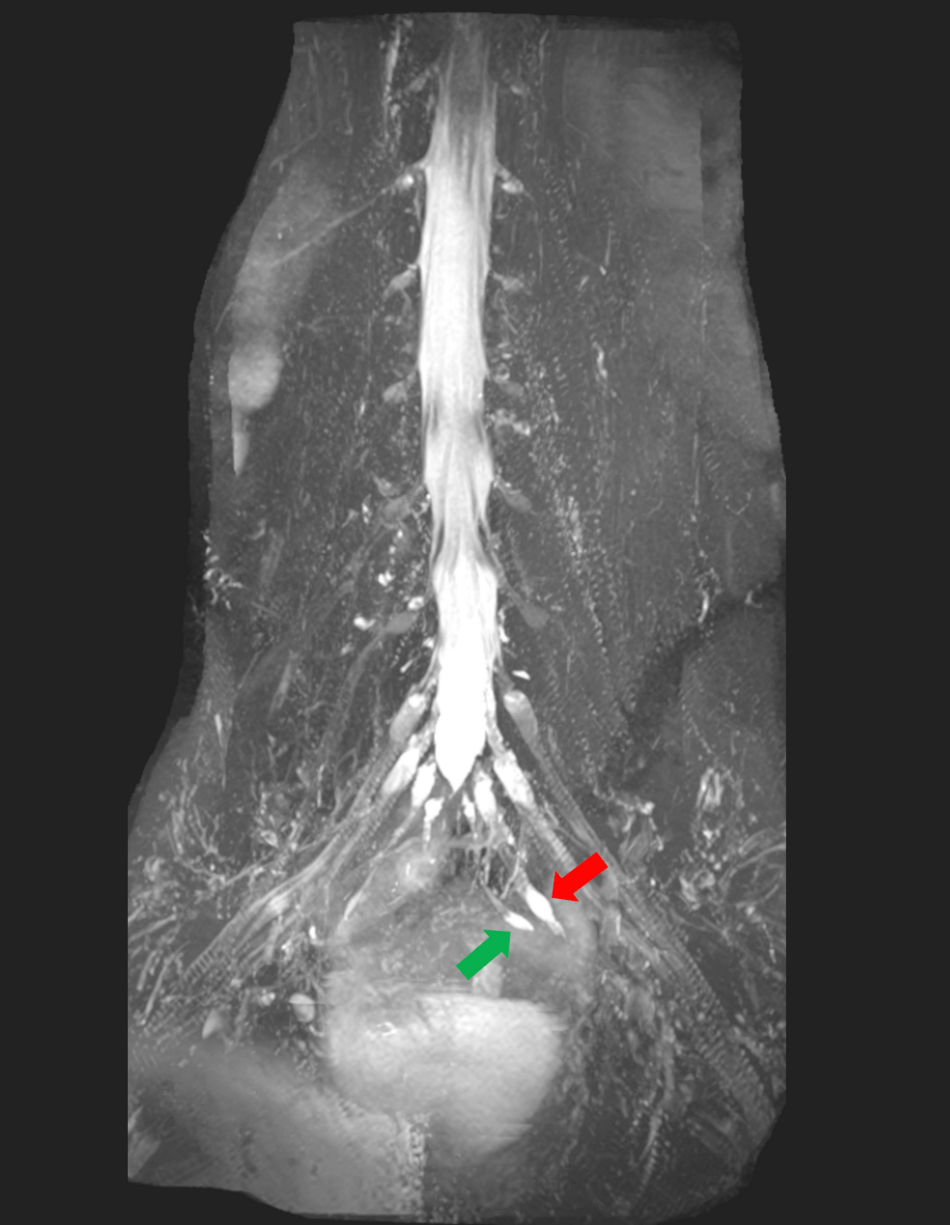
Nodular thickening and hyperintensity in the left-sided sacral plexus involving the left S3 (red arrow) and S4 (green arrow) nerve roots highlighted on maximum intensity projection of coronal view magnetic resonance imaging.

MRI whole spine, CT Head and CT thorax, abdomen and pelvis with contrast positively identified a subtle STIR hyperintense signal in bilateral lumbosacral plexus (Figure 1), possibly indicating mild oedema/inflammation, and nodular thickening and hyperintensity in the left-sided sacral plexus involving the left S3 and S4 nerve roots (Figure 2) that could represent small neuromas or could be post-inflammatory.

Differential diagnosis

At presentation, the initial differential diagnosis with acute onset bilateral lower limb weakness and reduced tendon reflex with recent vaccination history included GBS/acute inflammatory demyelinating polyneuropathy, transverse myelitis, radiculomyelitis and spinal cord compression.

Treatment

She was started on IVIG treatment at 0.4 grams per kilogram per day for suspected GBS after a lumbar puncture. This treatment was continued for a total of five days.

Outcome and follow-up

She remained an inpatient under the neurology team for 16 days. Throughout her inpatient stay, her observations remained stable and were within normal ranges on discharge.

Her functional status on discharge did not return to baseline. She was able to stand and mobilise 50 meters with a four-wheeled walker.

She has an EMG/NCS and neurology clinic follow-up with a planned repeat MRI lumbosacral plexus.

## Discussion

Vaccination programmes have enabled wide-spread protective active immunity against a range of microorganisms. However, as with any medical intervention, there are risks associated including neurological conditions such as GBS.

The first reported cases of GBS post-vaccination were identified in 1976 post-H1N1 swine flu vaccine. This led to an eight-fold increase in reported GBS cases [12]. However, further evaluation established the vaccine’s protective benefits outweighed the risk of developing GBS [13]. The COVID-19 vaccination programme saw a rise in cases with adenoviral vectored vaccines but overall, there was no increased risk of GBS [14]. Further studies have suggested a real but minimal risk of GBS post-vaccination [15].

The recombinant zoster vaccine (Shingrix, GSK) was licensed by the European Union in 2018 with clinical studies indicating high vaccine efficacy in people aged 50 years and above [16-18]. Initially, GBS was identified as a potential adverse side-effect, but the insufficient data could not conclude a safety problem and only warranted continued monitoring, with subsequent studies balancing the overall risk-benefits in favour of vaccination [19,20].

Published case reports of GBS post non-live recombinant vaccine against the varicella-zoster virus are summarised in Table 4. All cases were diagnosed after imaging and blood investigations excluded other neurological conditions. The age ranged between 61 and 76 years with two cases being men and two women. Two cases shared a common medical history of hypothyroidism with our case. The presentation was between 10 and 14 days post-Shingrex vaccination, which was slightly longer than the eight-day history of our case. Symptom presentation and examination findings were similar to our case with lower limb weakness and reduced sensation being the most prevalent among reported cases. Three cases were treated with IVIG and one with plasma exchange. None of the cases reached full resolution of symptoms but most showed marked improvement after therapy.

**Table 4:** Summary of literature search cases of post-vaccination GBS

Case and publication	Age, sex	Past medical history	Symptom onset	Presenting complaint	Examination	Blood investigation	CSF results	EMG result	Diagnosis	Management	Outcome
Case 1: Yadav et al. 2019 Journal of Neurology Neuroscience [8]	76, F	Hyperlipidemia, asthma, macular degeneration, pancreatitis	10 days, Shingrix (Recombinannt Zoster)	Bilateral proximal lower extremity weakness	Cranial nerves II-XII intact, 5/5 bilateral upper extremity strength, 5/5 bilateral lower dorsiflexion, Normal sensation bilaterally in upper and lower extremities, 3/5 hip flexion, Bilateral absent Patellar and Achilles tendon reflexes	N/A	Protein: 61 mg/dL, Leukocyte count: 0 CUMM, Cytoalbuminogenic dissociation	N/A	GBS	IVIG for five days followed by three plasma exchange treatments	The patient showed remarkable improvement with lower extremity strength and power.
Case 2: Zafar et al. 2020 RRNMF Neuromuscular Journal [9]	79, M	Clinically diagnosed neuropathy	10 days, Shingrix	Progressive tingling and numbness in feet	Diminished pinprick and vibration bilaterally from toes to the knees, Areflexia at biceps, brachioradialis, patella and Achilles, Romberg positive, broad-based gait, no loss of sensation in upper limbs. Weak foot dorsiflexion, hip flexion and knee extension bilaterally	N/A	Protein: 195 mg/dL, Leukocyte count: Negative	Demyelinating polyneuropathy	GBS	IVIG for 5 days	Improvement of strength with residual pinprick sensation deficit only in the toes and reduced vibration in his feet from his previous neuropathy two months after treatment.
Case 3: Wons et al. 2024 MDPI reports [10]	64, F	Tobacco usage, hypertension, hypothyroidism, insomnia, post-menopausal bleeding, urinary calculus	11 days, Shingrix vaccine	Fevers, numbness, tingling in her body, shuffling and stiff gait	N/A	Total protein: 8.2 g/dL, Glucose: 127 mg/dL, Alkaline phosphatase: 128IU/L, Haemoglobin: 11.4g/dL, Sedimentation rate: 43 mm/h, C-reactive protein: 1.1 mg/dL, Mean platelet volume: 12.6	Protein: 90 mg/dL, Leukocyte count: 452 CMM, Glucose: 76 mg/dL, Red blood cell count: 19 CMM	Normal	GBS with differential diagnoses including Lyme disease with atypical presentation, primary lymphomatous meningitis, CNS leukemia, or a paraneoplastic syndrome.	IVIG and Rocephin	Symptoms mostly subsided with occasional numbness in her feet and groin.
Case 4: Chohan et al. 2022 Cureus [11]	61, M	Hypothyroidism, bipolar disorder, obstructive sleep apnea, hyperlipidemia, lumbar spine surgery, right total knee replacement, AMSAN variant of GBS 10 months prior	14 days, second dose of Shingrix	Unsteadiness, Reduced distal upper and lower limb sensation, Shortness of beath	Pinprick sensation reduced globally, Vibration sensation reduced bilaterally below ankles, Impaired proprioception bilaterally below ankles, Absent deep tendon reflexes in bilateral upper and lower extremities	N/A	Protein: 84 mg/dL, Leukocyte count: 4 mm^3^, Glucose: 75 mg/dL, Lactic acid: 17 mg/dL, Oligoclonal bands: Negative	N/A	Acute recurrent exacerbation of the AMSAN variant of GBS.	Four sessions of PLEX therapy	Complete improvement in motor strength. Sensation and reflexes improved but not to baseline.

Our described case fits into the age demographic of similarly published case reports summarised in Table 4. The timeframe of symptom onset post-vaccination was shorter in our case compared to the literature cases, but the presenting symptoms have many similarities [8-11].

The leading molecular mimicry hypothesis behind the pathological mechanism of GBS requires further in vitro experiments to establish possible antigenic components of vaccinations including the Shingrix vaccine [7].

## Conclusions

Our case report and literature search illustrate that GBS is an uncommon condition especially in the post-vaccination population. However, the diagnosis should be considered as a differential in patients presenting with acute ascending bilateral lower limb neurology in the post-vaccination period including the Shingrix vaccine, especially in people who do not have the commonly recognised risk factors for GBS.

Furthermore, this report underscores the challenges associated with diagnosing and managing GBS, highlighting the importance of disease awareness in facilitating early diagnosis and timely initiation of treatment. Lastly, continued research to establish a broader understanding of GBS and its disease mechanism is necessary to help mitigate the disease incidence with future vaccine programmes.

## References

[1] van Doorn PA, Van den Bergh PY, Hadden RD (2023). European Academy of Neurology/Peripheral Nerve Society Guideline on diagnosis and treatment of Guillain-Barré syndrome. Eur J Neurol.

[2] Wachira VK, Farinasso CM, Silva RB, Peixoto HM, de Oliveira MR (2023). Incidence of Guillain-Barré syndrome in the world between 1985 and 2020: a systematic review. Glob Epidemiol.

[3] Bragazzi NL, Kolahi AA, Nejadghaderi SA (2021). Global, regional, and national burden of Guillain-Barré syndrome and its underlying causes from 1990 to 2019. J Neuroinflammation.

[4] Jee Y (2020). WHO International Health Regulations Emergency Committee for the COVID-19 outbreak. Epidemiol Health.

[5] Naeem S, Shabbir A, Khan AS, Ahmad S, Mustafa KJ, Fahim A (2016). Guillain-Barre syndrome following oral polio vaccination. J Neurovirol.

[6] Perez-Vilar S, Hu M, Weintraub E (2021). Guillain-Barré syndrome after high-dose influenza vaccine administration in the United States, 2018-2019 season. J Infect Dis.

[7] Israeli E, Agmon-Levin N, Blank M, Chapman J, Shoenfeld Y (2012). Guillain-Barré syndrome--a classical autoimmune disease triggered by infection or vaccination. Clin Rev Allergy Immunol.

[8] Yadav R, Hundley D, Cation L (2019). Severe Guillain-Barré syndrome following Shingrix® vaccine administration. J Neurol Neurosci.

[9] Zafar W, Aslam H, Digala L, Govindarajan R (2020). Shingrix vaccine and Guillain-Barre syndrome: a case report: vaccine related neuromuscular disease. RRNMF Neuromusc J.

[10] Wons MJ, Vaghela A, Khalid A, Brooks BD (2024). Serious and progressive neuropathy presumably post-shingrix vaccination. Reports.

[11] Chohan S, Chohan A (2022). Recurrence of a rare subtype of Guillain-Barré syndrome following a second dose of the shingles vaccine. Cureus.

[12] Schonberger LB, Bregman DJ, Sullivan-Bolyai JZ (1979). Guillain-Barre syndrome following vaccination in the National Influenza Immunization Program, United States, 1976--1977. Am J Epidemiol.

[13] Babazadeh A, Mohseni Afshar Z, Javanian M (2019). Influenza vaccination and Guillain-Barré syndrome: reality or fear. J Transl Int Med.

[14] Censi S, Bisaccia G, Gallina S, Tomassini V, Uncini A (2024). Guillain-Barré syndrome and COVID-19 vaccination: a systematic review and meta-analysis. J Neurol.

[15] Principi N, Esposito S (2019). Vaccine-preventable diseases, vaccines and Guillain-Barre' syndrome. Vaccine.

[16] Cunningham AL, Lal H, Kovac M (2016). Efficacy of the herpes zoster subunit vaccine in adults 70 years of age or older. N Engl J Med.

[17] Lal H, Cunningham AL, Godeaux O (2015). Efficacy of an adjuvanted herpes zoster subunit vaccine in older adults. N Engl J Med.

[18] López-Fauqued M, Campora L, Delannois F (2019). Safety profile of the adjuvanted recombinant zoster vaccine: pooled analysis of two large randomised phase 3 trials. Vaccine.

[19] Tavares-Da-Silva F, Co MM, Dessart C (2020). Review of the initial post-marketing safety surveillance for the recombinant zoster vaccine. Vaccine.

[20] Goud R, Lufkin B, Duffy J (2021). Risk of Guillain-Barré syndrome following recombinant zoster vaccine in Medicare beneficiaries. JAMA Intern Med.

